# Person Re-Identification Using Local Relation-Aware Graph Convolutional Network

**DOI:** 10.3390/s23198138

**Published:** 2023-09-28

**Authors:** Yu Lian, Wenmin Huang, Shuang Liu, Peng Guo, Zhong Zhang, Tariq S. Durrani

**Affiliations:** 1 Tianjin Key Laboratory of Wireless Mobile Communications and Power Transmission, Tianjin Normal University, Tianjin 300387, China; 2 CATARC (Tianjin) Automotive Engineering Research Institute Co., Ltd., Tianjin 300300, China; 3 Department of Electronic and Electrical Engineering, University of Strathclyde, Glasgow G1 1QE, UK

**Keywords:** person re-identification, graph convolutional network, local feature relationship

## Abstract

Local feature extractions have been verified to be effective for person re-identification (re-ID) in recent literature. However, existing methods usually rely on extracting local features from single part of a pedestrian while neglecting the relationship of local features among different pedestrian images. As a result, local features contain limited information from one pedestrian image, and cannot benefit from other pedestrian images. In this paper, we propose a novel approach named Local Relation-Aware Graph Convolutional Network (LRGCN) to learn the relationship of local features among different pedestrian images. In order to completely describe the relationship of local features among different pedestrian images, we propose overlap graph and similarity graph. The overlap graph formulates the edge weight as the overlap node number in the node’s neighborhoods so as to learn robust local features, and the similarity graph defines the edge weight as the similarity between the nodes to learn discriminative local features. To propagate the information for different kinds of nodes effectively, we propose the Structural Graph Convolution (SGConv) operation. Different from traditional graph convolution operations where all nodes share the same parameter matrix, SGConv learns different parameter matrices for the node itself and its neighbor nodes to improve the expressive power. We conduct comprehensive experiments to verify our method on four large-scale person re-ID databases, and the overall results show LRGCN exceeds the state-of-the-art methods.

## 1. Introduction

Person re-identification (re-ID) aims at matching pedestrians with the same identity across multiple camera views [1,2,3,4]. It has gained attention in recent years due to its wide range of video surveillance applications, including tracking suspects and locating missing individuals.

**Figure 1:**
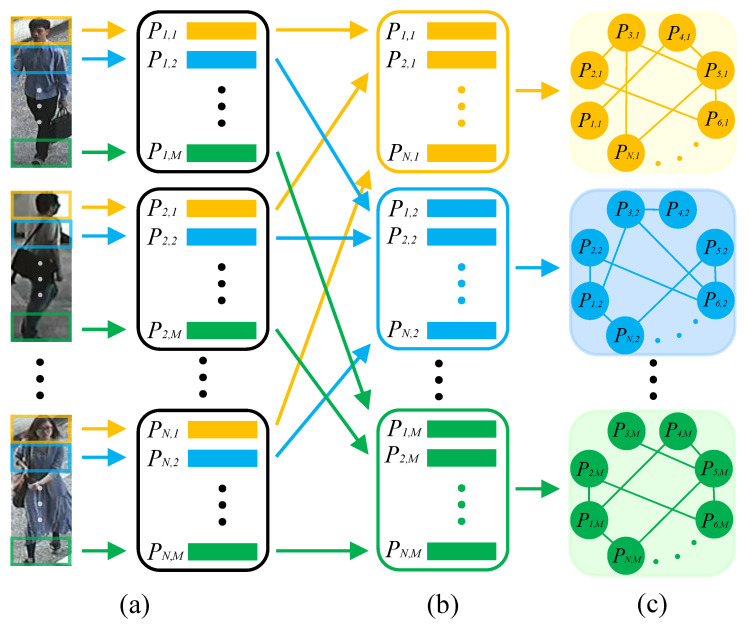
(**a**) In traditional person re-ID approaches, the extraction of local features is limited to a single part of the pedestrian. (**b**,**c**) The proposed LRGCN constructs graphs using local features from the same part of different pedestrian images to learn the relationship of local features among different pedestrian images.

With the renaissance of deep learning, Convolutional Neural Network (CNN) dominates the field of identity recognition, such as person re-ID [5,6,7,8] and gait recognition [9,10]. As for person re-ID, many CNN-based methods [11,12,13,14] focus on extracting global features of pedestrians, but they ignore the fine-grained information of pedestrians which is significant to distinguish the pedestrians, with high visual similarity. Local feature extraction has been proven to be an effective way to improve the feature representation in many research fields [15,16,17]. As for person-re-ID, some methods [18,19,20] divide the pedestrian image or convolutional activation maps into several horizontal parts. Subsequently, local features are extracted from these parts, as shown in Figure 1a. These methods achieve impressive performance in most public person re-ID databases. However, they only extract the local feature from single part of a pedestrian, which neglects the relationship of local features among different pedestrian images. As a result, these local features contain the limited information extracted from one pedestrian image, and cannot benefit from other pedestrian images.

Recently, Graph Convolutional Network (GCN) has attracted significant attention due to its effectiveness on graph data processing [21,22]. They propagate the information of nodes over the graph structure, and therefore, the nodes of graph aggregate the information from other nodes. Hence, it is reasonable that we resort to GCN to establish the relationship of local features among different pedestrian images. However, we should take the following two aspects into consideration for the person re-ID task. The one is how to construct the graph for local features, and the other is how to propagate the information for different kinds of nodes effectively.

In this paper, we propose a novel approach named Local Relation-Aware Graph Convolutional Network (LRGCN) to solve the above-mentioned questions for person re-ID. Specifically, a pedestrian image is divided into several parts based on key points to overcome misalignment. Then, each part’s local features are extracted. Since the same parts of different pedestrian images, especially different images with the same ID, could describe the pedestrian from the different aspects, we can learn the complementary information after building the relationship among them. Thus, to solve the first question, we construct the graphs using local features from the same part of a pedestrian, as depicted in Figure 1b,c.

Concretely, in order to completely describe the relationship among local features from the same part of different pedestrian images, we propose an overlap graph and similarity graph. As for the overlap graph, the local features from the same part are treated as the nodes. The two nodes with the same ID are prone to have more common nodes in their neighborhoods; these common nodes are denoted as overlap nodes, and they should be assigned to a larger edge weight. Hence, we formulate the edge weight of the overlap graph as the overlap node number in the node’s neighborhoods. In this way, the overlap graph is robust to environmental variations due to considering the contexts of nodes, and the nodes in the overlap graph could learn the information from other nodes accurately.

The similarity graph considers local features from the same part as nodes, and regards the similarity between nodes as edge weight. In order to improve the flexibility of the similarity graph, we learn two different transformations to measure the similarity between nodes. As a consequence, the adjacency matrix of similarity graph is asymmetric and data-driven. Furthermore, we update the graph topology of similarity graph in each graph convolutional layer, so it is more flexible than the heuristic predefined graph structure. The discriminative ability of local features is enhanced through the propagation of node information in the similarity graph.

After constructing the graphs, they should be fed into the graph convolution layer, but most graph convolution operations share the same parameter matrix for all the nodes, which is hard to discover interaction of among the nodes. Hence, to propagate the information for different kinds of nodes effectively (i.e., the second question), we propose the Structural Graph Convolution (SGConv). In the aggregation stage, a node is updated depending on itself and its neighbor nodes, and therefore, these nodes are naturally divided into the node itself and its neighbor nodes. Inspired by this, the proposed SGConv learns different parameter matrices for the two types of nodes so as to improve the expressive power of GCN. The proposed SGConv is concise and it is applicable to arbitrary graph topologies.

To summarize, our contributions include the following three aspects:

(1) We propose LRGCN, a person re-ID method, that considers the relationship between local features across different pedestrian images so as to learn valuable information from other pedestrian images.
(2) We design an overlap graph and similarity graph to model the relationship of local features among different pedestrian images from different aspects. Based on the two kinds of graphs, we could obtain robust and discriminative local features.
(3) We propose SGConv, which learns different parameter matrices for the node itself and its neighbor nodes to improve the expressive power of GCN.

The effectiveness of each component in LRGCN is validated through rigorous ablation experiments. Meanwhile, our method outperforms state-of-the-art methods on four large-scale person re-ID databases. Furthermore, we also present visualization results of overlap graph and similarity graph, which demonstrates the effectiveness of our method qualitatively.

## 2. Related Work

### 2.1. Person Re-ID

Benefiting from the multi-layer non-linear structure of CNN, pedestrian images are represented by discriminative deep features. Li et al. [23] and Zhao et al. [24] were the first to apply CNN to person re-ID and achieve great success. Subsequently, many researchers have designed various CNN models to learn feature representations of pedestrian images to improve the feature discrimination, an idea borrowed from other fields [9,25]. As for person re-ID, some of these methods [26,27,28] primarily concentrate on learning global features. For example, Yi et al. [26] utilized a structurally symmetric Siamese-CNN to directly learn the similarity between pedestrian images. Yang et al. [27] presented the Class Activation Maps Augmentation (CAMA) for person re-ID, which designs multiple branches to mine complementary visual information from entire pedestrian images. Wei et al. [28] proposed the Self-Inspirited Feature Learning (SIF) for person re-ID, which improves the discriminative ability of given models using a negative branch.

Some approaches learn local features to provide fine-grained information of pedestrian. Zhao et al. [29] implemented the localization of pedestrian body parts and local feature learning in a unified framework of deep network. Zhang et al. [30] proposed to align local features by calculating the shortest path between them in order to overcome occlusion and pose variation. Park et al. [31] introduced a relation network for person re-ID, which exploits a one-vs.-rest relational module to inject the local features from other parts into the representations.

To further alleviate the influences of pose variations, background clutter, and misalignment, an increasing number of approaches introduce external clues, e.g., human pose estimation [32,33,34], in their models. Su et al. [35] proposed the Pose-driven Deep Convolutional (PDC) model that applies pose transformation in the learning process of local features to overcome pose variations. Huang et al. [36] divided convolutional activation maps based on human pose estimation and extracted local features from the aligned parts. Kalayeh et al. [19] designed the SPReID model for person re-ID, which replaces the rectangular bounding boxes with human semantic parsing so as to precisely localize the body parts with arbitrary contours. Tay et al. [37] proposed the Attribute Attention Network (AANet) that constructs attribute attention maps to obtain strong discriminative representations using additional attribute information.

In addition, some works try to improve the accuracy of person re-ID using gallery images, such as manifold learning [38,39] and re-ranking [40]. Loy et al. [38] propagated query image label information among gallery images in an unsupervised manner to obtain robust ranking results. Bai et al. [39] proposed the Supervised Smoothed Manifold (SSM), which leverages the training data label constraint to learn a smooth similarity measure. Zhong et al. [40] utilized *k*-reciprocal encoding to optimize the ranking list. These methods belong to post-processing and pedestrian image features cannot be improved from these post-processing operations. Recently, Luo et al. [41] proposed Spectral Feature Transformation (SFT) which employs the relationship among images to optimize group-wise similarities. However, SFT has no learnable parameters, so it cannot model the relationship among images accurately. In contrast to the previously mentioned methods, our method can adaptively learn the relationship among pedestrian images using two types of graphs to improve the feature representation capacity.

### 2.2. Graph Convolutional Network

CNN has achieved great success in dealing with Euclidean structure data, but it cannot be directly applied to non-Euclidean structure data, i.e., graph structure data [21,42,43]. However, lots of critical data, such as knowledge graphs and social networks, can be represented using graph structures. Recently, GCN has been proposed to learn graph structure data and has achieved impressive performance [44,45,46,47]. GCN is usually constructed from spectral perspective and spatial perspective. Spectral-based methods [21,46] perform the convolution operation using the graph Fourier transform in the frequency domain. As for spatial-based methods [48,49,50], they expand the convolution filter to graph structure data in order to implement the convolution operation among nodes. It is noteworthy that our work follows the spatial perspective to construct GCN.

In some recent work [51,52,53,54], GCN has been introduced into person re-ID. Furthermore, ref. [53] achieved satisfactory improvement when applying GCN to global features. This indicates that it is effective to use GCN to construct the relationship among pedestrian images so as to improve feature representations. However, global features neglect to emphasize local differences and lack explicit mechanisms to effectively address the issue of misalignment. Meanwhile, we observe that using a single graph, e.g., [53], to construct the relationship among pedestrian images is suboptimal. Therefore, we extend GCN to local features and propose overlap graph and similarity graph to fully explore the relationship of local features among different pedestrian images. Besides, we also propose SGConv, which learns different parameter matrices for the node itself and its neighbor nodes to improve the expressiveness of GCN.

## 3. Approach

**Figure 2:**
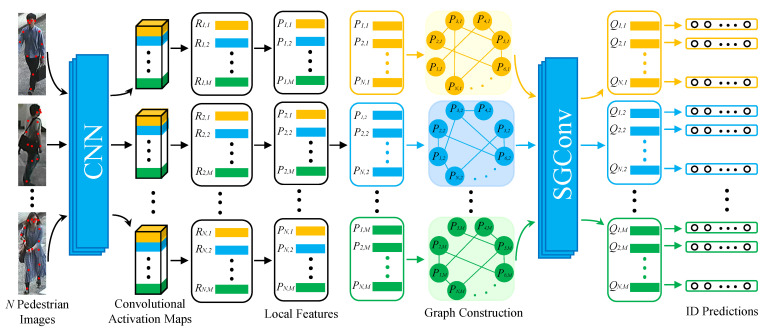
The framework of the proposed LRGCN. We first utilize the CNN model to extract the local features $R_{n,m}$ of the pedestrian, and then $R_{n,m}$ is followed by a convolutional layer with the kernel size of 1×1 to obtain the dimension-reduced local features $P_{n,m}$. Afterwards, we construct the graphs taking $P_{n,m}$ as the nodes and perform SGConv on the graphs to learn the relationship of local features among different pedestrian images. Finally, $Q_{n,m}$ is fed into the classifier to conduct the ID predictions.

In this section, we introduce the proposed LRGCN, and its framework is shown in Figure 2. Firstly, we present the process of extracting local features. Then, we detail the overlap graph, the similarity graph, and SGConv on the two kinds of graphs.

### 3.1. Extraction of Local Features

To extract the local features of a pedestrian, we first resize the pedestrian images into 384×128. These resized images are then input into the CNN model of LRGCN. The CNN model is implemented by ResNet-50 [55], where we remove the down-sampling operation of Conv5_1 and the fully connected layer FC-1000. Then, we obtain the convolutional activation maps with the size of 2048×24×8, where 2048 is the number of channels, and 24 and 8 are the height and width of convolutional activation map, respectively. Meanwhile, to overcome the misalignment, we locate 17 key points of pedestrians using the pose estimator [56]. Afterwards, we divide the convolutional activation maps into *M* parts based on these key points and extract the local feature $R_{n,m}$∈R${}^{2048\times 1}$(n=1,2,⋯,N and m=1,2,⋯,M) from each part via max pooling [36]. Here, *N* is the number of pedestrian images, $R_{n,m}$ indicates the local feature of the *m*-th part in the *n*-th pedestrian image, and *M* is set to 9 as [36]. Finally, $R_{n,m}$ is followed by a convolutional layer with the kernel size of 1×1 to obtain the dimension-reduced local feature $P_{n,m}\in R^{512\times 1}$.

### 3.2. Learning Relationship among Local Features

**Figure 3:**
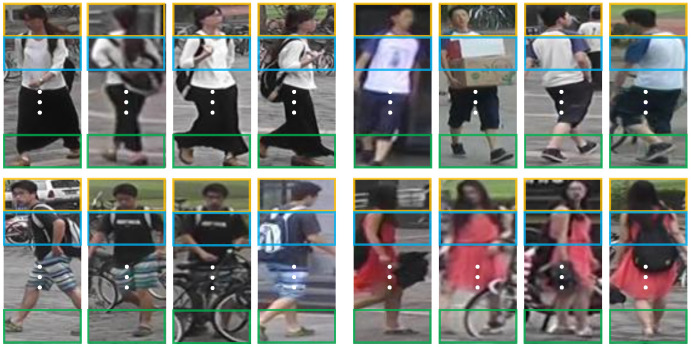
Different pedestrian images from the same identity.

Our motivation is to learn the relationship of local features among different pedestrian images so as to improve the feature representation capacity. However, what kind of local relationship is conducive to feature representation? From Figure 3, we can see that different pedestrian images possess different appearances. That is, the same parts of pedestrian images provide different perspectives to describe the pedestrian. Based on the above observation, we expect to learn the relationship among local features from the same parts to obtain the complementary information, thereby improving the representation ability of features. Meanwhile, with the help of graph convolution operation, the nodes in a graph can send its information to other nodes and receive information from other nodes to learn from each other. Hence, for the same part of different pedestrian images, we resort to the graph to establish the relationship among them. In order to describe the relationship completely, we design two types of graphs from different aspects, namely, the overlap graph and similarity graph. In these graphs, the nodes are represented by the local features from the same parts, and the edge weight reflects the connection strength between nodes. As a result, with the node information propagation over graphs, the complementary information could be integrated into local features effectively.

**Overlap Graph.** For the overlap graph, the nodes are represented by the local features $P_{n,m}$. Since the nodes with the same ID usually have more overlapping nodes in their neighborhoods, we define the overlap node number in the node’s neighborhoods as the edge weight. Specifically, for the local features from the *m*-th parts in pedestrian images, we propose the adjacency matrix $O^{m}$$=[o_{i,j}^{m}]$∈R${}^{N\times N}$ to represent the relationship among them, and $o_{i,j}^{m}$ represents the edge weight between the *m*-th parts in the *i*-th and the *j*-th pedestrian images. It is defined as:

(1) $$o_{i,j}^{m}=\left\{\begin{array}{c} |\delta \left(P_{i,m},k\right)\cap \delta \left(P_{j,m},k\right)|,\;i\neq j\\ 0,\;i=j \end{array}\right.$$

where $\delta (P_{i,m},k)$ and $\delta (P_{j,m},k)$ are the sets of *k* nearest neighborhoods of $P_{i,m}$ and $P_{j,m}$, respectively, ∩ indicates the intersection of two sets, and |·| represents the element number of a set. From Equation (1) we can see that $o_{i,j}^{m}$ is the overlap node number of *k* nearest neighborhoods of $P_{i,m}$ and $P_{j,m}$ when i≠j.

We expect the nodes with the same ID to have larger edge weights, which is beneficial to learn complementary information. Therefore, the distribution of local features $P_{n,m}$ and the selection of *k* nearest neighborhoods are important for overlap graph construction. Therefore, the cross-entropy loss is applied to optimize the distribution of $P_{n,m}$ so that the nodes with the same ID are closer to each other. Furthermore, since the concatenated local feature of a pedestrian image is generally more robust than a single local feature, we select the *k* nearest neighborhoods based on the Euclidean distance between the concatenated local features of two nodes. The distance between two nodes is formulated as:

(2) $$\begin{array}{c} D\left(P_{i,m},P_{j,m}\right)={∥P_{i,\cdot }-P_{j,\cdot }∥}_{2} \end{array}$$

(3) $$\begin{array}{c} P_{i,\cdot }=\langle P_{i,1},P_{i,2},\cdots ,P_{i,m},\cdots ,P_{i,M}\rangle \end{array}$$

(4) $$\begin{array}{c} P_{j,\cdot }=\langle P_{j,1},P_{j,2},\cdots ,P_{j,m},\cdots ,P_{j,M}\rangle \end{array}$$

where 〈·〉 represents the vector concatenation. Based on Equations (2)–(4), we can find that $\delta \left(P_{i,1},k\right)=\delta \left(P_{i,2},k\right)=\cdots =\delta \left(P_{i,M},k\right)$ because we utilize the concatenated local feature to replace the single local feature when computing *k* nearest neighborhoods, so $O^{1}=O^{2}=\cdots =O^{M}$. In other words, we do not need to repeatedly construct the adjacency matrix of overlap graph for local features from different parts of the pedestrian.

Finally, we normalize $O^{m}$:

(5) $$\begin{array}{c} O^{m^{\prime }}=\Lambda^{-\frac{1}{2}}O^{m}\Lambda^{-\frac{1}{2}}+I \end{array}$$

where Λ is a diagonal matrix and $\Lambda_{i,i}$ = $\sum_{j}$$o_{i,j}^{m}$ indicates the *i*-th diagonal element of Λ. In addition, *I* is an identity matrix, and it sets the edge weight of the node itself to 1 to prevent the over-smoothing.

**Figure 4:**
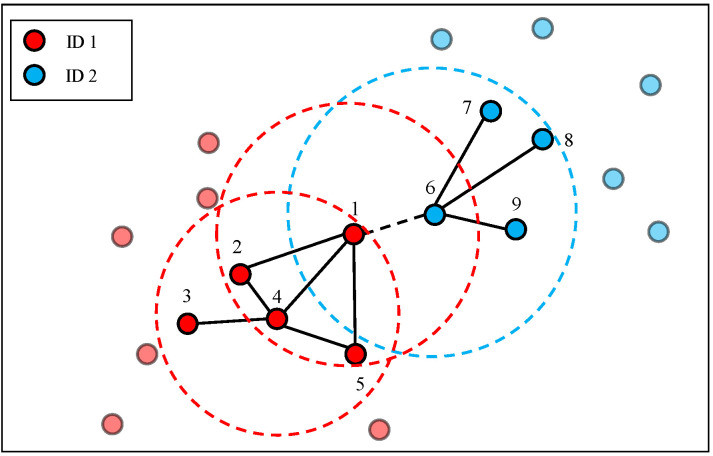
An example of edge weight calculation in the overlap graph.

For ease of understanding, as depicted in Figure 4, we give an example of edge weight calculation. If we assume k=4, then δ(node 1, 4) = {node 2, node 4, node 5, node 6}, δ(node 4, 4) = {node 1, node 2, node 3, node 5}, and δ(node 6, 4) = {node 1, node 7, node 8, node 9}. Consequently, |δ(node 1, 4) ∩δ(node 4, 4)| = |{node 2, node 5}| = 2, that is, the edge weight between node 1 and node 4 is 2. |δ(node 1, 4) ∩δ(node 6, 4)| = |Ø| = 0, that is, the edge weight between node 1 and node 6 is 0. Here, we find an interesting phenomenon. Nodes with the same ID (node 1 and node 4) have larger edge weight than nodes with different IDs (node 1 and node 6), even though node 6 is closer to node 1 than node 4. It is clear that the overlap graph is robust to environmental variations because the contexts of nodes are considered.

**Similarity Graph.** To enhance the flexibility of deep model, we design the similarity graph to describe the relationship of local features among different pedestrian images. The similarity graph treats the local features $P_{n,m}$ as the nodes, and takes the similarity between nodes as the edge weight. Specifically, for the local features from the *m*-th parts in pedestrian images, their relationship is described by the adjacency matrix $S^{m}=\left[s_{i,j}^{m}\right]\in R^{N\times N}$, and $s_{i,j}^{m}$ represents the edge weight between the *m*-th parts in the *i*-th and the *j*-th pedestrian images. It is defined as:

(6) $$\begin{array}{c} s_{i,j}^{m}=softmax\left(\varphi {\left(P_{i,m}\right)}^{T}\psi \left(P_{j,m}\right)\right)=\frac{e^{\varphi {\left(P_{i,m}\right)}^{T}\psi \left(P_{j,m}\right)}}{\sum_{n=1}^{N}e^{\varphi {\left(P_{i,m}\right)}^{T}\psi \left(P_{n,m}\right)}} \end{array}$$

where φ and ψ are two transformation functions and they are performed using the convolutional layer with the kernel size of 1×1. The adjacency matrix of the similarity graph could improve the flexibility of the similarity graph because it is asymmetric and data-driven. Note that weak edge weights may be the noise, so we set the edge weights from less than 0.01 to 0.

It is worthy of note that for multi-layer graph convolutional network, the similarity graph is reconstructed in each layer according to the nodes of corresponding layer. In other words, we update the graph topology of similarity graph in each graph convolutional layer, which further enhances the flexibility of similarity graph.

**Structural Graph Convolution.** In the traditional graph convolutional layers [21,44,49], its input is the adjacency matrix $A\in R^{N\times N}$ and the node feature matrix $X\in R^{d_{i}\times N}$. Here, $d_{i}$ represents the node dimension and *N* is the number of nodes. Then, the node feature matrix is updated by propagating the node information in the graph. The convolution operation is formulated as:

(7) $$\begin{array}{c} Y=\mu (WXA) \end{array}$$

where *W*∈R${}^{d_{o}\times d_{i}}$ is the parameter matrix, $d_{0}$ is the node dimension after updating, and μ(·) is a non-linear activation function.

Following Equation (7), the graph convolutional operation can be decomposed into three steps. Firstly, by left multiplying *X* by *W*, node representations are transformed using a learnable parameter matrix. Secondly, by right multiplying (WX) by *A*, the node collects transformed information from itself and its neighbor nodes. Finally, μ(·) is applied to conduct a non-linear transformation. In the first step, all the nodes of graph are treated equally, and they share the same parameter matrix, which is hard to discover complex interaction of the nodes. In the second step, since the transformed information is from the node itself and its neighbor nodes, we naturally divide the nodes into two categories and utilize different parameter matrices on them. Hence, an improved graph convolution operation called SGConv is proposed, which is formulated as:

(8) $$\begin{array}{c} Y=\mu (W_{0}X\left(I⊙A\right)+W_{1}X\left(\left(1-I\right)⊙A\right)) \end{array}$$

where ⊙ represents the element-wise multiplication, *I* is an identity matrix, 1 is a matrix with all elements of 1, and $W_{0}$ and $W_{1}$ are the parameter matrices for the node itself and its neighbor nodes, respectively.

In LRGCN, for the *m*-th parts of pedestrian images, we set $A=O^{m^{\prime }}\;+S^{m}$ and $X=X^{m}$, and Equation (8) is reformulated as:

(9) $$\begin{array}{cc} Y^{m}= & \mu (W_{0}^{m}X^{m}\left(I⊙\left(O^{m^{\prime }}\;+S^{m}\right)\right)+W_{1}^{m}X^{m}(\left(1-I\right)⊙\\ & \left(O^{m^{\prime }}\;+S^{m})\right)),\;m\in \left\{1,2,\cdots ,M\right\} \end{array}$$

where $X^{m}$∈R${}^{d_{i}\times N}$ consists of local features from the *m*-th parts in pedestrian images, $W_{0}^{m}$∈R${}^{d_{o}\times d_{i}}$ and $W_{1}^{m}$∈R${}^{d_{o}\times d_{i}}$ are the parameter matrices, and μ(·) is implemented by the ReLU function. For the first SGConv layer, $X^{m}=\left[P_{1,m},P_{2,m},\cdots ,P_{N,m}\right]$.

**Figure 5:**
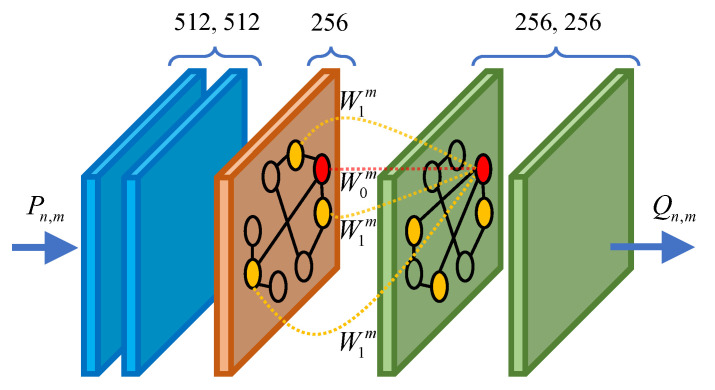
The SGConv layers in LRGCN.

In this work, we design five SGConv layers for LRGCN, as shown in Figure 5. The output feature dimension of each SGConv layer is 512, 512, 256, 256, and 256, respectively. The output of the last layer is denoted as $Q_{n,m}$, and then $Q_{n,m}$ is fed into a Softmax classifier to predict the identity probability and calculate the cross-entropy loss. The Softmax classifier is formulated as:

(10) $$\begin{array}{c} {\hat{H}}_{n,m}=softmax\left(Q_{n,m}V\right) \end{array}$$

where *V* is the parameters of classifier implemented by a fully connected layer, ${\hat{H}}_{n,m}=\left[{\hat{h}}_{n,m}^{t}\right]\in R^{T\times 1}$ is the predicted identity vector, ${\hat{h}}_{n,m}^{t}$ represents the predicted probability that $Q_{n,m}$ belongs to the *t*-th identity, and *T* is the number of pedestrian identities.

The cross-entropy loss of $Q_{n,m}$ is formulated as:

(11) $$\begin{array}{c} L_{n,m}=\sum_{t=1}^{T}-h_{n,m}^{t}log\left({\hat{h}}_{n,m}^{t}\right) \end{array}$$

where $h_{n,m}^{t}$ indicates the true probability that $Q_{n,m}$ belongs to the *t*-th identity. If $Q_{n,m}$ belongs to the *z*-th identity, then $h_{n,m}^{z}=1$; otherwise $h_{n,m}^{t}$ = 0.

Finally, the total loss is formulated as:

(12) $$\begin{array}{c} L_{total}=\sum_{n=1}^{N}\sum_{m=1}^{M}L_{n,m}. \end{array}$$

## 4. Experiments

In this section, we first introduce person re-ID databases and implementation details. Afterwards, we conduct an ablation study and report the results of the proposed LRGCN compared with state-of-the-art methods. Finally, we analyze the influence of several hyperparameters for LRGCN and visualize the overlap graph and the similarity graph.

### 4.1. Databases

**Market-1501** [57] contains 32,668 images of 1501 identities. According to the database setting, the training set consists of 12,936 images with 751 identities. The test set consists of 19,732 images with 750 identities.

**DukeMTMC-reID** [58] comprises of 36,411 images of 1404 identities, 16,522 images from 702 identities for training, 19,889 images from other 702 identities for testing. Both the training set and the test set contain 702 non-overlapping identities.

**CUHK03** [24] contains 14,097 pedestrian images of 1467 identities, and each identity is observed by one of five camera pairs. We utilize the same setting as [27,36,40,59], where the training set includes 767 identities and the test set includes 700 identities. CUHK03 provides two kinds of bounding boxes, i.e., DPM-detected and hand-labeled. We choose the DPM-detected bounding boxes that are closer to the realistic setting.

**MSMT17** [60] comprises 126,441 images captured by 15 cameras. Meanwhile, it is also the most challenging database for person re-ID due to significant changes in scene, lighting, viewpoint, and pose. There are 32,621 images of 1041 identities in the training set and 93,820 images of 3060 identities in the test set.

**Figure 6:**
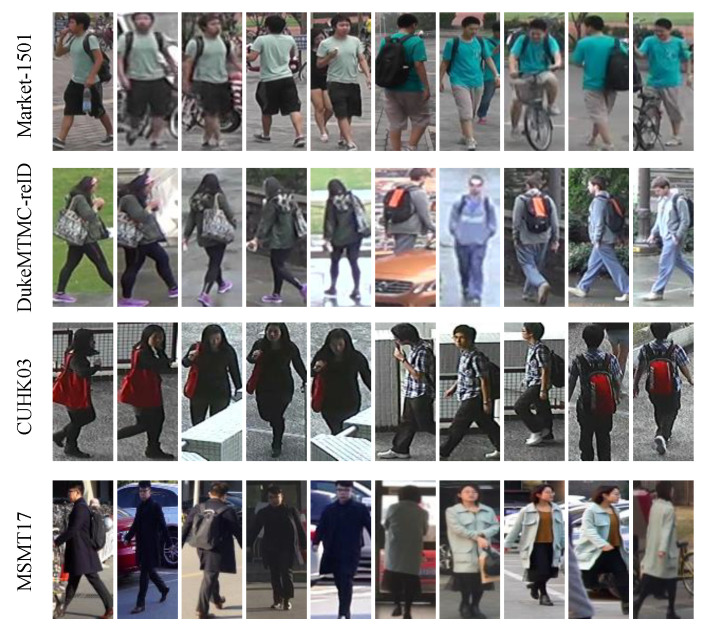
Some images from four databases.

Some pedestrian images from the four person re-ID databases are presented in Figure 6.

### 4.2. Implementation Details

We resize all the pedestrian images to 384×128 and utilize random horizontal flipping and random cropping for data augmentation. The batch size is set to 66 during training. We first randomly select 11 identities from the training set, and then randomly choose 6 pedestrian images for each identity. We utilize SGD optimizer with momentum to train LRGCN, where the momentum is 0.9 and the weight decay is $5\times 10^{-4}$.

### 4.3. Ablation Experiments

We perform ablation experiments on four databases so as to investigate the contribution of each component in LRGCN. As shown in Table 1, CNN denotes that we only utilize the CNN model of LRGCN to learn feature representations, and CNN + re-ranking denotes that we use re-ranking technology for CNN. CNN + *S* employ the similarity graph to model the relationship of local features among different pedestrian images, and CNN + S_sharing adopts the same transformation function to process $P_{i,m}$ and $P_{j,m}$, i.e., φ = ψ in Equation (6). CNN + *O* employ the overlap graph to model the relationship of local features among different pedestrian images, CNN + O_single adopts the single local feature to replace the concatenated local feature when selecting the *k* nearest neighborhoods for the overlap graph, and CNN + O_updating indicates that we update the overlap graph in each graph convolutional layer. CNN + *O* + *S* denotes that SGConv is replaced by the traditional graph convolution operation, and LRGCN_concatenating denotes that we apply GCN to the concatenated local features.

From Table 1, several conclusions can be drawn. Firstly, CNN + *S* and CNN + *O* significantly exceed CNN due to the consideration of the relationship of local features among different pedestrian images. Specifically, CNN + *O* improve CNN in mAP from 84.1%, 73.0%, 66.8%, and 52.8% to 87.6% (+3.5%), 76.3% (+3.3%), 72.8% (+6.0%), and 58.2% (+5.4%) on the four databases, respectively. Secondly, CNN + *S* outperforms CNN + S_sharing. This is because using different transformation functions for $P_{i,m}$ and $P_{j,m}$ could improve the flexibility of similarity graph. Thirdly, compared with CNN + O_single, CNN + *O* gains higher rank-1 accuracy and mAP on the four databases. This indicates that the concatenated local feature of pedestrian image is more robust than single local feature when selecting *k* nearest neighborhoods. Fourthly, the performance of CNN + *O* and CNN + O_updating is similar. Therefore, we do not update the overlap graph to reduce computation cost. Fifthly, CNN + *O* + *S* improves the performance of CNN + *S* and CNN + *O* after combining the overlap graph and the similarity graph. This is because the two kinds of graphs can describe the relationship of local features among different pedestrian images from different aspects. Sixthly, LRGCN achieves better performance than CNN + *O* + *S* due to SGConv treating node itself and its neighbor nodes differently. Seventhly, LRGCN clearly exceeds LRGCN_concatenating. It shows that applying GCN to local features is more effective than concatenated local features. Finally, although the re-ranking technique significantly improve CNN performance, our method surpasses CNN_re-ranking in rank-1 accuracy by +1.4%, +2.2%, +2.6%, and +1.3% on the four databases. Re-ranking is a post-processing method and does not improve the feature representations. In our method, the feature representations can benefit from the relationship learned by GCN and we do not adopt the re-ranking technique for the results calculated based on GCN features.

### 4.4. Comparison with State-of-the-Art Approaches

**Market-1501.** As depicted in Table 2, we report mAP of 90.7% and on rank-1 accuracy of 96.5%, which exceeds the performance of all previous methods. Compared with EANet [36], the proposed LRGCN raises 6.2% and 2.1% on mAP and rank-1 accuracy. Although EANet also utilize 17 key points to divide the convolutional activation maps, it ignores the relationship of local features among different pedestrian images. However, our method models the relationship of local features among different pedestrian images via constructing two kinds of graphs and using SGConv to propagate useful information, so that the local features of pedestrian images could learn complementary information from each other.

**DukeMTMC-reID.** In Table 3, the proposed LRGCN achieves the best performance. The proposed LRGCN significantly exceeds SGGNN [53] by 11.8% for mAP and 9.5% for rank-1 accuracy. This is because SGGNN discovers the relationship of global features among different pedestrian images using one kind of graph, while the proposed LRGCN constructs two kinds of graphs from different aspects to model the relationship of local features among different pedestrian images.

**CUHK03.** The comparison results on the CUHK03 database are listed in Table 4. The proposed LRGCN obtains mAP of 75.3% and rank-1 accuracy of 76.1%, which significantly outperforms all the compared methods. RNet-S [31] combines the information of different parts of the same pedestrian image, while our method learns the relationship among the same parts of different pedestrian images. Hence, LRGCN improves RNet-S [31] by 5.8% and 3.6% for mAP and rank-1 accuracy.

**MSMT17.** In Table 5, the proposed LRGCN reports mAP of 60.6% and rank-1 accuracy of 82.7%. Compared with Auto-ReID [68], our method achieves +8.1% on mAP and +4.5% on rank-1 accuracy. The results indicate that the proposed LRGCN is beneficial for a more realistic and challenging person re-ID database.

### 4.5. Parameter Analysis

We analyze the effect of *k* in Equation (1) and the effect of SGConv layer number on the four databases.

**Figure 7:**
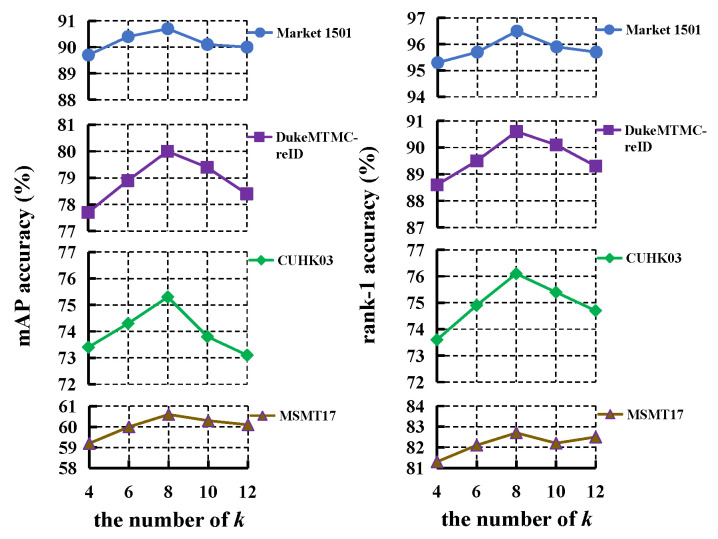
The effect of *k* for LRGCN.

Firstly, we evaluate the effect of *k* in Equation (1), which is related to the adjacency matrix of overlap graph, and the results are presented in Figure 7. The mAP and rank-1 improves all the four databases when *k* increases from 4 to 8, while both of them decline when k>8. Therefore, we choose k=8 in the experiments.

**Figure 8:**
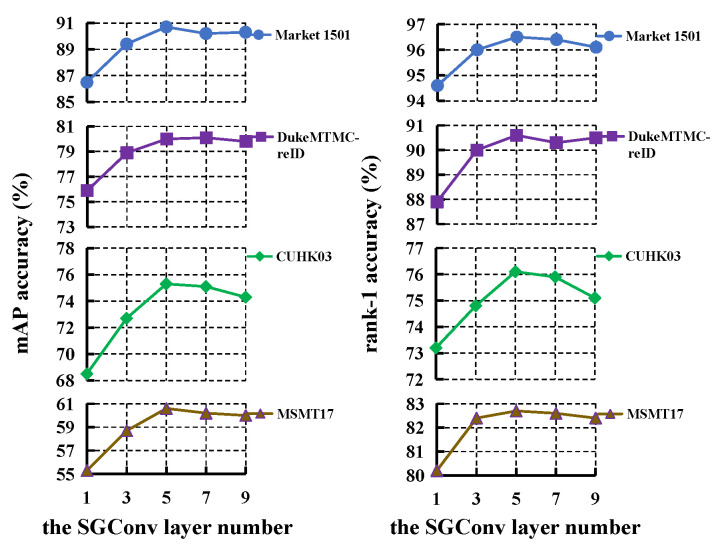
The effect of SGConv layer number for LRGCN.

Secondly, we study the effect of the SGConv layer number for LRGCN. As depicted in Figure 8, the performance rises as the number of SGConv layer increases. Deeper GCN allows nodes to learn more abstract information from each other, which is beneficial to improving the expressive power of features. However, the performance reaches saturation when the number of SGConv layer is larger than 5. Hence, we set the number of SGConv layer to 5 on the four databases.

### 4.6. Time Analysis

Table 6 lists the inference time of each query image in CNN (baseline) and LRGCN, where *q* denotes the number of query images and *g* denotes the number of gallery images. From Table 6, the inference time of CNN and LRGCN increases with the increase of the gallery image number. This is because larger number of gallery images requires more time cost for retrieval. Besides, both CNN and LRGCN meet the requirements of real-time application on all databases expect for MSMT17.

### 4.7. Visualization

**Figure 9:**
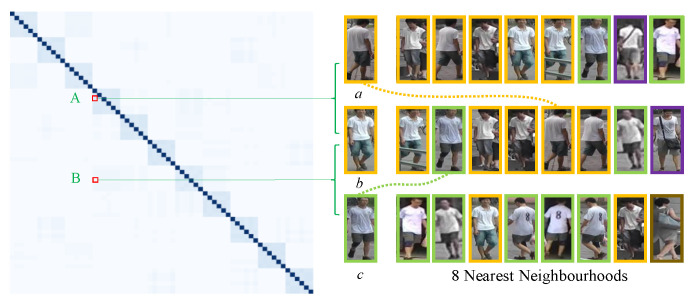
Visualization of the adjacency matrix of the overlap graph. A and B represent the edge weights between images *a* and *b*, and images *b* and *c* respectively.

Figure 9 shows an example of the adjacency matrix of overlap graph. In the left color matrix, each color block corresponds to an element in the adjacency matrix, and the deeper color represents the larger value. For each image, we list its 8 nearest neighborhoods and use the same color bounding boxes to mark the pedestrian images with the same identity.

As can be seen from Figure 9, *a* ranks 5th in the 8 nearest neighborhoods of *b*, but they are with the same ID and the overlap node number between them is 4. In contrast, *c* ranks 2nd in the 8 nearest neighborhoods of *b*, but they have different IDs and the overlap node number between them is 2. In short, as for the overlap graph, nodes with the same ID have larger edge weight than the nodes with different IDs, even if the nodes with different IDs are closer. This verifies the robustness of overlap graph to environmental variations.

**Figure 10:**
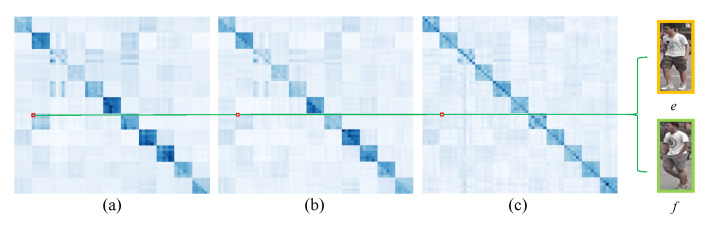
Visualization of the adjacency matrix of the similarity graph. The (**a**–**c**) correspond to the adjacency matrices of similarity graphs in the 1st, 3rd, and 5th SGConv layers, respectively. The *e* and *f* denote pedestrian images with different IDs, respectively.

The visualization of the adjacency matrix of similarity graph is illustrated in Figure 10 where (a), (b), and (c) correspond to the adjacency matrices of similarity graphs in the 1st, 3rd, and 5th SGConv layers, respectively. Obviously, the adjacency matrices of these similarity graphs are asymmetric and different. This shows that each SGConv layer can learn different similarity graphs. Meanwhile, we find that although images *e* and *f* with different IDs have large edge weight in the 1st similarity graph, the edge weight between them gradually decreases in the 3rd and 5th similarity graph. This indicates that building unique similarity graph for each layer can correct some unreasonable edge weights in the learning process so that nodes can effectively learn complementary information.

In the comparison of Figure 9 and Figure 10, we can find that the adjacency matrices of the overlap graph and similarity graph are different because they establish the relationship of local features among different pedestrian images from different aspects. Hence, when combining the similarity graph and the overlap graph, the performance is further improved.

## 5. Conclusions

In this paper, we have proposed LRGCN to learn the relationship of local features among different pedestrian images. Specifically, we have constructed two kinds of graphs, i.e., an overlap graph and similarity graph, to fully mine the relationship of local features among different pedestrian images. Moreover, we have proposed SGConv, which treats the node itself and its neighbor nodes differently to effectively propagate information in the graph. As a result, we have obtained robust and discriminative local features. We have fully verified LRGCN on four large-scale person re-ID databases, and the experimental results have shown that our method surpasses the state-of-the-art methods.

## Figures and Tables

**Table 1:** Ablation experiments on Market-1501, DukeMTMC-reID, CUHK03, and MSMT17.

Methods	Market-1501		DukeMTMC-reID		CUHK03		MSMT17	
	mAP (%)	Rank-1 (%)	mAP (%)	Rank-1 (%)	mAP (%)	Rank-1 (%)	mAP (%)	Rank-1 (%)
CNN	84.1	94.0	73.0	85.6	66.8	72.1	52.8	78.5
CNN + re-ranking	89.9	95.1	82.7	88.4	74.7	73.5	58.2	81.4
CNN + *S*_sharing	86.0	94.7	74.5	86.1	69.1	72.5	57.2	79.7
CNN + *S*	86.6	95.0	75.2	86.9	70.7	73.0	57.6	80.1
CNN + *O*_single	86.9	95.3	75.1	87.5	72.5	73.4	58.0	80.7
CNN + *O*_updating	87.7	95.3	76.6	88.4	72.6	73.9	58.1	80.3
CNN + *O*	87.6	95.5	76.3	88.2	72.8	73.6	58.2	81.0
CNN + *S* + *O*	89.0	95.9	78.1	89.0	73.9	74.6	59.1	81.5
LRGCN_concatenating	88.0	94.4	76.7	87.3	71.4	72.9	56.5	78.1
LRGCN	**90.7**	**96.5**	**80.0**	**90.6**	**75.3**	**76.1**	**60.6**	**82.7**

**Table 2:** Comparison on the Market-1501 database.

Methods	Market-1501	
	mAP (%)	Rank-1 (%)
BoW + kissme [57]	20.8	44.4
MFFM (HOG + LBP) [61]	-	70.1
MGCAM [62]	74.3	83.8
AOS [63]	70.4	86.5
DaRe [64]	76.0	89.0
MLFN [65]	74.3	90.0
HA-CNN [66]	75.7	91.2
SGGNN [53]	82.8	92.3
PCB [18]	77.3	92.4
Mancs [59]	82.3	93.1
GCSL [51]	81.6	93.5
EANet [36]	84.5	94.4
IANet [67]	83.1	94.4
Auto-ReID [68]	85.1	94.5
CAMA [27]	84.5	94.7
DG-Net [69]	86.0	94.8
CDPM [70]	86.0	95.2
RNet-S [31]	88.0	94.8
OSNet [71]	86.7	94.8
ICA [72]	82.3	93.3
AGW + DA + Joint [73]	88.6	95.2
LRGCN (Ours)	**90.7**	**96.5**

**Table 3:** Comparison on the DukeMTMC-reID database.

Methods	DukeMTMC-reID	
	mAP (%)	rank-1 (%)
BoW + kissme [57]	12.2	25.1
AOS [63]	62.1	79.2
DaRe [64]	64.5	80.2
HA-CNN [66]	63.8	80.5
MLFN [65]	62.8	81.0
SGGNN [53]	68.2	81.1
PCB [18]	65.3	81.9
GCSL [51]	69.5	84.9
Mancs [59]	71.8	84.9
CAMA [27]	72.9	85.8
EANet [36]	73.3	86.1
DG-Net [69]	74.8	86.6
IANet [67]	73.4	87.1
CDPM [70]	77.5	88.2
RNet-S [31]	77.1	89.3
OSNet [71]	76.6	88.7
ICA [72]	71.6	85.6
LRGCN (Ours)	**80.0**	**90.6**

**Table 4:** Comparison on the CUHK03 database.

Methods	CUHK03	
	mAP (%)	Rank-1 (%)
BoW + kissme [57]	6.4	6.4
HA-CNN [66]	38.6	41.7
MGCAM [62]	46.9	46.7
AOS [63]	43.3	47.1
MLFN [65]	47.8	52.8
PCB [18]	54.2	61.3
DaRe [64]	59.0	63.3
Mancs [59]	60.5	65.5
CAMA [27]	64.2	66.6
EANet [36]	66.2	72.0
Auto-ReID [68]	69.3	73.3
CDPM [70]	67.0	71.9
RNet-S [31]	69.5	72.5
OSNet [71]	67.8	72.3
ICA [72]	59.3	64.6
AGW + DA + Joint [73]	69.2	70.3
LRGCN (Ours)	**75.3**	**76.1**

**Table 5:** Comparison on the MSMT17 database.

Methods	MSMT17	
	mAP (%)	Rank-1 (%)
IANet [67]	46.8	75.5
DG-Net [69]	52.3	77.2
Auto-ReID [68]	52.5	78.2
OSNet [71]	55.1	79.1
AGW + DA + Joint [73]	50.0	68.2
LRGCN (Ours)	**60.6**	**82.7**

**Table 6:** Comparison of inference time between LRGCN and CNN (baseline) on four databases.

Methods	Market-1501		DukeMTMC-reID		CUHK03		MSMT17	
	q:3368	g:15913	q:2228	g:17661	q:1400	g:5332	q:11659	g:82161
	ms	fps	ms	fps	ms	fps	ms	fps
CNN	13	77	15	67	5	200	73	14
LRGCN (Ours)	22	45	25	40	12	83	112	9

## Data Availability

The databases used to train and evaluate our method are publicly available [24,57,58,60].

## References

[1] Lin R., Wang R., Zhang W., Wu A., Bi Y. (2023). Joint Modal Alignment and Feature Enhancement for Visible-Infrared Person Re-Identification. Sensors.

[2] Zhou Y., Liu P., Cui Y., Liu C., Duan W. (2022). Integration of Multi-Head Self-Attention and Convolution for Person re-identification. Sensors.

[3] Zhou J., Dong Q., Zhang Z., Liu S., Durrani T.S. (2023). Cross-Modality Person Re-Identification via Local Paired Graph attention network. Sensors.

[4] Yu H.X., Zheng W.S. Weakly Supervised Discriminative Feature Learning with State Information for Person Identification. Proceedings of the IEEE/CVF Conference on Computer Vision and Pattern Recognition (CVPR).

[5] Song W., Li S., Chang T., Hao A., Zhao Q., Qin H. (2020). Context-Interactive CNN for Person Re-Identification. IEEE Trans. Image Process..

[6] Varior R.R., Haloi M., Wang G. Gated Siamese Convolutional Neural Network Architecture for Human Re-identification. Proceedings of the European Conference on Computer Vision (ECCV).

[7] Shen Y., Li H., Xiao T., Yi S., Chen D., Wang X. Deep Group-Shuffling Random Walk for Person Re-Identification. Proceedings of the IEEE/CVF Conference on Computer Vision and Pattern Recognition (CVPR).

[8] Shen Y., Xiao T., Yi S., Chen D., Wang X., Li H. (2021). Person Re-Identification with Deep Kronecker-Product Matching and Group-Shuffling Random Walk. IEEE Trans. Pattern Anal. Mach. Intell..

[9] Wu Z., Huang Y., Wang L., Wang X., Tan T. (2017). A Comprehensive Study on Cross-View Gait Based Human Identification with Deep CNNs. IEEE Trans. Pattern Anal. Mach. Intell..

[10] Khan M.H., Farid M.S., Grzegorzek M. (2020). A Non-Linear View Transformations Model for Cross-View Gait Recognition. Neurocomputing.

[11] Xiao T., Li H., Ouyang W., Wang X. Learning Deep Feature Representations with Domain Guided Dropout for Person Re-Identification. Proceedings of the IEEE/CVF Conference on Computer Vision and Pattern Recognition (CVPR).

[12] Ahmed E., Jones M., Marks T.K. An Improved Deep Learning Architecture for Person Re-Identification. Proceedings of the IEEE/CVF Conference on Computer Vision and Pattern Recognition (CVPR).

[13] Chen W., Chen X., Zhang J., Huang K. A Multi-Task Deep Network for Person Re-Identification. Proceedings of the AAAI Conference on Artificial Intelligence.

[14] Liu J., Ni B., Yan Y., Zhou P., Cheng S., Hu J. Pose Transferrable Person Re-Identification. Proceedings of the IEEE/CVF Conference on Computer Vision and Pattern Recognition (CVPR).

[15] Wang H., Kläser A., Schmid C., Liu C.L. (2013). Dense Trajectories and Motion Boundary Descriptors for Action Recognition. Int. J. Comput. Vis..

[16] Zheng L., Huang Y., Lu H., Yang Y. (2019). Pose-Invariant Embedding for Deep Person Re-Identification. IEEE Trans. Image Process..

[17] Yao H., Zhang S., Hong R., Zhang Y., Xu C., Tian Q. (2019). Deep Representation Learning with Part Loss for Person Re-Identification. IEEE Trans. Image Process..

[18] Sun Y., Zheng L., Yang Y., Tian Q., Wang S. Beyond Part Models: Person Retrieval with Refined Part Pooling (and A Strong Convolutional Baseline). Proceedings of the European Conference on Computer Vision (ECCV).

[19] Kalayeh M.M., Basaran E., Gökmen M., Kamasak M.E., Shah M. Human Semantic Parsing for Person Re-Identification. Proceedings of the IEEE/CVF Conference on Computer Vision and Pattern Recognition (CVPR).

[20] Miao J., Wu Y., Liu P., Ding Y., Yang Y. Pose-Guided Feature Alignment for Occluded Person Re-Identification. Proceedings of the IEEE/CVF International Conference on Computer Vision (ICCV).

[21] Kipf T.N., Welling M. (2016). Semi-Supervised Classification with Graph Convolutional Networks. arXiv.

[22] Zhou J., Cui G., Hu S., Zhang Z., Yang C., Liu Z., Wang L., Li C., Sun M. (2020). Graph neural networks: A review of methods and applications. AI Open.

[23] Li W., Zhao R., Xiao T., Wang X. DeepReID: Deep Filter Pairing Neural Network for Person Re-Identification. Proceedings of the IEEE/CVF Conference on Computer Vision and Pattern Recognition (CVPR).

[24] Zhao R., Ouyang W., Wang X. Learning Mid-Level Filters for Person Re-identification. Proceedings of the IEEE/CVF Conference on Computer Vision and Pattern Recognition (CVPR).

[25] Leng L., Li M., Kim C., Bi X. (2017). Dual-Source Discrimination Power Analysis for Multi-Instance Contactless Palmprint Recognition. Multimed. Tools Appl..

[26] Yi D., Lei Z., Liao S., Li S.Z. Deep Metric Learning for Person Re-identification. Proceedings of the 22nd International Conference on Pattern Recognition.

[27] Yang W., Huang H., Zhang Z., Chen X., Huang K., Zhang S. Towards Rich Feature Discovery with Class Activation Maps Augmentation for Person Re-Identification. Proceedings of the IEEE/CVF Conference on Computer Vision and Pattern Recognition (CVPR).

[28] Wei L., Wei Z., Jin Z., Yu Z., Huang J., Cai D., He X., Hua X.S. (2020). SIF: Self-Inspirited Feature Learning for Person Re-Identification. IEEE Trans. Image Process..

[29] Zhao L., Li X., Zhuang Y., Wang J. Deeply-Learned Part-Aligned Representations for Person Re-Identification. Proceedings of the IEEE/CVF International Conference on Computer Vision (ICCV).

[30] Zhang X., Luo H., Fan X., Xiang W., Sun Y., Xiao Q., Jiang W., Zhang C., Sun J. (2017). Alignedreid: Surpassing Human-Level Performance in Person Re-Identification. arXiv.

[31] Park H., Ham B. Relation Network for Person Re-Identification. Proceedings of the AAAI Conference on Artificial Intelligence.

[32] Wei S.E., Ramakrishna V., Kanade T., Sheikh Y. Convolutional Pose Machines. Proceedings of the IEEE/CVF Conference on Computer Vision and Pattern Recognition (CVPR).

[33] Shelhamer E., Long J., Darrell T. (2017). Fully Convolutional Networks for Semantic Segmentation. IEEE Trans. Pattern Anal. Mach. Intell..

[34] Cao Z., Simon T., Wei S.E., Sheikh Y. Realtime Multi-Person 2D Pose Estimation Using Part Affinity Fields. Proceedings of the IEEE/CVF Conference on Computer Vision and Pattern Recognition (CVPR).

[35] Su C., Li J., Zhang S., Xing J., Gao W., Tian Q. Pose-Driven Deep Convolutional Model for Person Re-Identification. Proceedings of the IEEE/CVF International Conference on Computer Vision (ICCV).

[36] Huang H., Yang W., Chen X., Zhao X., Huang K., Lin J., Huang G., Du D. (2018). EANet: Enhancing Alignment for Cross-Domain Person Re-Identification. arXiv.

[37] Tay C.P., Roy S., Yap K.H. AANet: Attribute Attention Network for Person Re-Identifications. Proceedings of the IEEE/CVF Conference on Computer Vision and Pattern Recognition (CVPR).

[38] Loy C.C., Liu C., Gong S. Person Re-Identification by Manifold Ranking. Proceedings of the IEEE International Conference on Image Processing.

[39] Bai S., Bai X., Tian Q. Scalable Person Re-Identification on Supervised Smoothed Manifold. Proceedings of the IEEE/CVF Conference on Computer Vision and Pattern Recognition (CVPR).

[40] Zhong Z., Zheng L., Cao D., Li S. Re-Ranking Person Re-Identification with *k*-Reciprocal Encoding. Proceedings of the IEEE/CVF Conference on Computer Vision and Pattern Recognition (CVPR).

[41] Luo C., Chen Y., Wang N., Zhang Z. Spectral Feature Transformation for Person Re-Identification. Proceedings of the IEEE/CVF International Conference on Computer Vision (ICCV).

[42] Bruna J., Zaremba W., Szlam A., LeCun Y. (2013). Spectral Networks and Locally Connected Networks on Graphs. arXiv.

[43] Veličković P., Cucurull G., Casanova A., Romero A., Liò P., Bengio Y. (2017). Graph Attention Networks. arXiv.

[44] Shi L., Zhang Y., Cheng J., Lu H. Two-Stream Adaptive Graph Convolutional Networks for Skeleton-Based Action Recognition. Proceedings of the IEEE/CVF Conference on Computer Vision and Pattern Recognition (CVPR).

[45] Chen Z.M., Wei X.S., Wang P., Guo Y. Multi-Label Image Recognition with Graph Convolutional Networks. Proceedings of the IEEE/CVF Conference on Computer Vision and Pattern Recognition (CVPR).

[46] Defferrard M., Bresson X., Vandergheynst P. Convolutional Neural Networks on Graphs with Fast Localized Spectral Filtering. Proceedings of the Advances in Neural Information Processing Systems.

[47] Mohamed A., Qian K., Elhoseiny M., Claudel C. Social-STGCNN: A Social Spatio-Temporal Graph Convolutional Neural Network for Human Trajectory Prediction. Proceedings of the IEEE/CVF Conference on Computer Vision and Pattern Recognition (CVPR).

[48] Yan S., Xiong Y., Lin D. Spatial Temporal Graph Convolutional Networks for Skeleton-Based Action Recognition. Proceedings of the AAAI Conference on Artificial Intelligence.

[49] Wang Z., Zheng L., Li Y., Wang S. Linkage Based Face Clustering via Graph Convolution Network. Proceedings of the IEEE/CVF Conference on Computer Vision and Pattern Recognition (CVPR).

[50] Niepert M., Ahmed M., Kutzkov K. Learning Convolutional Neural Networks for Graphs. Proceedings of the 33rd International Conference on Machine Learning.

[51] Chen D., Xu D., Li H., Sebe N., Wang X. Group Consistent Similarity Learning via Deep CRF for Person Re-Identification. Proceedings of the IEEE/CVF Conference on Computer Vision and Pattern Recognition (CVPR).

[52] Yan Y., Zhang Q., Ni B., Zhang W., Xu M., Yang X. Learning Context Graph for Person Search. Proceedings of the IEEE/CVF Conference on Computer Vision and Pattern Recognition (CVPR).

[53] Shen Y., Li H., Yi S., Chen D., Wang X. Person Re-identification with Deep Similarity-Guided Graph Neural Network. Proceedings of the European Conference on Computer Vision (ECCV).

[54] Wu Y., Bourahla O.E.F., Li X., Wu F., Tian Q., Zhou X. (2020). Adaptive Graph Representation Learning for Video Person Re-Identification. IEEE Trans. Image Process..

[55] He K., Zhang X., Ren S., Sun J. Deep Residual Learning for Image Recognition. Proceedings of the IEEE/CVF Conference on Computer Vision and Pattern Recognition (CVPR).

[56] Xiao B., Wu H., Wei Y. Simple Baselines for Human Pose Estimation and Tracking. Proceedings of the European Conference on Computer Vision (ECCV).

[57] Zheng L., Shen L., Tian L., Wang S., Wang J., Tian Q. Scalable Person Re-Identification: A Benchmark. Proceedings of the IEEE/CVF International Conference on Computer Vision (ICCV).

[58] Ristani E., Solera F., Zou R., Cucchiara R., Tomasi C. Performance Measures and a Data Set for Multi-target, Multi-camera Tracking. Proceedings of the European Conference on Computer Vision (ECCV).

[59] Wang C., Zhang Q., Huang C., Liu W., Wang X. Mancs: A Multi-task Attentional Network with Curriculum Sampling for Person Re-identification. Proceedings of the European Conference on Computer Vision (ECCV).

[60] Wei L., Zhang S., Gao W., Tian Q. Person Transfer GAN to Bridge Domain Gap for Person Re-Identification. Proceedings of the IEEE/CVF Conference on Computer Vision and Pattern Recognition (CVPR).

[61] Wang S., Xu X., Liu L., Tian J. (2020). Multi-level feature fusion model-based real-time person re-identification for forensics. J. Real-Time Image Process..

[62] Song C., Huang Y., Ouyang W., Wang L. Mask-Guided Contrastive Attention Model for Person Re-Identification. Proceedings of the IEEE/CVF Conference on Computer Vision and Pattern Recognition (CVPR).

[63] Huang H., Li D., Zhang Z., Chen X., Huang K. Adversarially Occluded Samples for Person Re-Identification. Proceedings of the IEEE/CVF Conference on Computer Vision and Pattern Recognition (CVPR).

[64] Wang Y., Wang L., You Y., Zou X., Chen V., Li S., Huang G., Hariharan B., Weinberger K.Q. Resource Aware Person Re-Identification Across Multiple Resolutions. Proceedings of the IEEE/CVF Conference on Computer Vision and Pattern Recognition (CVPR).

[65] Chang X., Hospedales T.M., Xiang T. Multi-Level Factorisation Net for Person Re-Identification. Proceedings of the IEEE/CVF Conference on Computer Vision and Pattern Recognition (CVPR).

[66] Li W., Zhu X., Gong S. Harmonious Attention Network for Person Re-Identification. Proceedings of the IEEE/CVF Conference on Computer Vision and Pattern Recognition (CVPR).

[67] Hou R., Ma B., Chang H., Gu X., Shan S., Chen X. Interaction-and-Aggregation Network for Person Re-Identification. Proceedings of the IEEE/CVF Conference on Computer Vision and Pattern Recognition (CVPR).

[68] Quan R., Dong X., Wu Y., Zhu L., Yang Y. Auto-ReID: Searching for a Part-Aware ConvNet for Person Re-Identification. Proceedings of the IEEE/CVF Conference on Computer Vision and Pattern Recognition (CVPR).

[69] Zheng Z., Yang X., Yu Z., Zheng L., Yang Y., Kautz J. Joint Discriminative and Generative Learning for Person Re-Identification. Proceedings of the IEEE/CVF Conference on Computer Vision and Pattern Recognition (CVPR).

[70] Wang K., Ding C., Maybank S.J., Tao D. (2020). CDPM: Convolutional Deformable Part Models for Semantically Aligned Person Re-Identification. IEEE Trans. Image Process..

[71] Zhou K., Yang Y., Cavallaro A., Xiang T. (2022). Learning Generalisable Omni-Scale Representations for Person Re-Identification. IEEE Trans. Pattern Anal. Mach. Intell..

[72] Wang M., Ma H., Huang Y. (2023). Information Complementary Attention-Based Multidimension Feature Learning for Person Re-Identification. Eng. Appl. Artif. Intell..

[73] Lin X., Zhu L., Yang S., Wang Y. (2023). Diff Attention: A Novel Attention Scheme for Person Re-Identification. Comput. Vis. Image Underst..

